# Comparative Transcriptome Analysis Identifies Desmoglein-3 as a Potential Oncogene in Oral Cancer Cells

**DOI:** 10.3390/cells12232710

**Published:** 2023-11-26

**Authors:** Hong Wan, Muy-Teck Teh, Giulia Mastroianni, Usama Sharif Ahmad

**Affiliations:** 1 Center for Immunobiology and Regenerative Medicine, Institute of Dentistry, Barts and the London School of Medicine and Dentistry, Queen Mary University of London, London E1 2AT, UK; m.t.teh@qmul.ac.uk (M.-T.T.); u.ahmad@qmul.ac.uk (U.S.A.); 2 School of Biological and Behavioural Sciences, Faculty of Science and Engineering, Queen Mary University of London, London E1 4NS, UK; g.mastroianni@qmul.ac.uk

**Keywords:** desmoglein-3, oral squamous carcinoma cells, RNA-Seq, bioinformatics, transmission electron microscopy, desmosomes, MMP-13, GJA1

## Abstract

The role of desmoglein-3 (DSG3) in oncogenesis is unclear. This study aimed to uncover molecular mechanisms through comparative transcriptome analysis in oral cancer cells, defining potential key genes and associated biological processes related to DSG3 expression. Four mRNA libraries of oral squamous carcinoma H413 cell lines were sequenced, and 599 candidate genes exhibited differential expression between DSG3-overexpressing and matched control lines, with 12 genes highly significantly differentially expressed, including 9 upregulated and 3 downregulated. Genes with known implications in cancer, such as MMP-13, KRT84, OLFM4, GJA1, AMOT and ADAMTS1, were strongly linked to DSG3 overexpression. Gene ontology analysis indicated that the DSG3-associated candidate gene products participate in crucial cellular processes such as junction assembly, focal adhesion, extracellular matrix formation, intermediate filament organisation and keratinocyte differentiation. Validation of RNA-Seq was performed through RT-qPCR, Western blotting and immunofluorescence analyses. Furthermore, using transmission electron microscopy, we meticulously examined desmosome morphology and revealed a slightly immature desmosome structure in DSG3-overexpressing cells compared to controls. No changes in desmosome frequency and diameter were observed between the two conditions. This study underscores intricate and multifaceted alterations associated with DSG3 in oral squamous carcinoma cells, implying a potential oncogenic role of this gene in biological processes that enable cell communication, motility and survival.

## 1. Introduction

Desmoglein 3 (DSG3) is a cell adhesion molecule that plays a crucial role in desmosome assembly. Desmosomes are specialized intercellular junctions found in epithelial tissues that anchor keratin intermediate filament and provide strong adhesion between cells and thus are the key to the maintenance of tissue structure and integrity. The assembly of desmosomes is a complex and coordinated process involving the synthesis, trafficking and interaction of various proteins, with DSG3 playing a central role in initiating desmosome formation through its homophilic and heterophilic interactions with itself and other desmosomal isoforms located on the neighbouring cell as well as with those on the same cells. Although DSG3 is known primarily to serve as a cell adhesion protein in desmosomes, mounting evidence suggests that it also participates in cell signalling processes beyond adhesion. For instance, DSG3 is involved in regulating indirectly the Wnt/β-catenin signalling pathway via its interaction with plakoglobin [1]. Increased DSG3 levels were shown to be correlated with reduced nuclear plakoglobin accompanied with elevated expression of the LEF/TCF transcriptional targets, cyclin D1, c-Myc and MMP7 in the tissues of head and neck cancer patients and oral squamous cell carcinoma lines [2,3,4]. DSG3 is also reported to participate in regulating kinases and phosphatases, and one example is its interaction with the Src kinase, which is involved in multiple signalling pathways that have an impact on cell proliferation, migration and survival [5,6,7]. Additionally, DSG3 has been shown to interact with various scaffolding proteins and signalling adaptors, such as plakoglobin and p0071, within desmosomes. These interactions can potentially mediate crosstalk between cell adhesion and signalling pathways, allowing DSG3 to influence signal transduction events. In particular, relevant to this study is that DSG3 can regulate epidermal growth factor receptor (EGFR), a receptor involved in cell growth, proliferation and survival, and regulate its signalling [8,9,10,11,12,13] and thus affect downstream pathways such as MAPK/ERK, PI3K/Akt and YAP [13,14,15]. It is important to note that the understanding of DSG3′s role in cell signalling is still evolving, and further research is needed to elucidate the precise mechanisms and functional implications of its involvement in signalling pathways.

Due to its important roles in cell biology, DSG3 has been implicated in various diseases and pathological conditions. The most well-known is pemphigus vulgaris, an autoimmune blistering disorder characterised by the production of autoantibodies against DSG3 and DSG1, another desmoglein isoform. The autoantibodies disrupt the interaction between desmogleins, leading to weakened cell–cell adhesion in the epidermis and mucous membranes, leading to the formation of skin blisters and mucosal erosions. DSG3 is also one of the autoantibody targets in paraneoplastic pemphigus, a rare autoimmune condition associated with malignancies and lymphoproliferative disorders often affecting multiple organ systems [16]. Another example is Hailey–Hailey disease, known as familial benign pemphigus and caused by mutations in the ATP2C1 gene that encodes for a calcium pump responsible for maintaining calcium homeostasis in keratinocytes [17,18]. Dysfunctional ATP2C1 leads to impaired desmosome function and reduced levels of DSG3, resulting in compromised cell–cell adhesion and the formation of persistent blisters and erosions.

Alterations in DSG3 expression have also been observed in squamous cell carcinoma (SCC) in multiple organ systems. DSG3 is often upregulated in SCCs, and its overexpression has been associated with increased tumour invasiveness and metastasis [2,14,19,20]. However, the role of DSG3 in cancer remains not fully understood, and contradictory findings have been reported in the literature [13,21,22,23]. For instance, a recent investigation discovered that contrary to expectations, DSG3 inhibits the collective cell migration in oral SCC cell lines [13]. Mechanistically, it was found that DSG3 limits the activity of the EGFR signalling pathway that in turn triggers the phosphorylation and subsequent export of YAP (yes-associated protein) from the cell nucleus, leading to its inactivation and inhibition of cell migration. Despite these findings, a comprehensive understanding of how DSG3 contributes to the regulation of gene expression in epithelial cells is still lacking. Using RNA-Seq, this study explored in-depth the role of DSG3 in the regulation of the transcriptome network in oral SCC cells. This study reveals that DSG3 is involved in various cellular processes, including extracellular matrix (ECM) organisation, focal adhesion, intercellular junction assembly and keratinocyte functions. Interestingly, the results indicate that DSG3 has a dual role. While it promotes intercellular junction assembly, it also somehow inhibits the association of desmosomes with keratin intermediate filaments, suggesting that DSG3 may exert a function in finely tuning cellular adhesion and keratinocyte maturation.

## 2. Materials and Methods

### 2.1. Cell Lines

Human oral SCC cell line H413 was obtained from Professor Prime’s lab [24]. Myc-tagged DSG3 was ectopically and stably expressed in the H413 cell line (D), which was used in a previous study [13]. The matched control cell line was H413 cells with transduction of empty vector (V). Both cell lines were cultured in growth medium, i.e., Dulbecco’s Modified Eagle Medium (DMEM, Lonza, Basel, Switzerland)/Ham’s F-12 (Thermo Fisher Scientific, Waltham, MA, USA) 1:1 supplemented with 10% (*v*/*v*) foetal bovine serum (FBS; Thermo Fisher Scientific) and 0.5 μg/mL hydrocortisone (Sigma, Dorset, UK) at 37 °C in a humidified atmosphere of 5% CO_2_/95% air. The medium was changed twice a week. Cells were passaged once a week. Cell stocks were thawed from liquid nitrogen and recovered in a growth medium for at least a couple of weeks before being used for experiments. For all experiments, cell lines were seeded and cultured in the growth medium. 

### 2.2. Sample Preparation

For RNA-Seq analysis, four samples of two conditions, i.e., empty vector control (V) and DSG3 transduced cell line (D), were prepared, each having two different passage numbers. Cell stocks from liquid nitrogen were thawed and recovered in culture with growth medium for at least a couple of weeks before cell counting and plating in a 12-well plate at confluent density (0.5 × 10^6^ cells/well). Cells reached confluence the next day and were manually scraped off from the wells, spun down and stored at −80 °C. Similar procedures were used for the extraction of cell lysates for RT-qPCR analysis with lysis buffer as described previously [13]. 

For transmission electron microscopy (TEM), H413-V and D cell lines were seeded at confluent density in a 6-well plate in duplicate, and after two days of culture, epithelial monolayers were established. Cells were washed briefly with PBS and treated with dispase (2.4 U/mL) for 20 min to detach the epithelial sheets from the substrate. They were washed briefly with phosphate buffer before fixation with freshly prepared 4% glutaraldehyde in phosphate buffer overnight. After washing with phosphate buffer, samples were stored in a fridge before being processed for TEM sample preparation and analysis by a specially trained technician. 

### 2.3. Extraction, RNA Library Preparation and NovaSeq Sequencing 

Total RNA was extracted from frozen cell pellets using a Qiagen RNeasy Mini kit following the manufacturer’s instructions (Qiagen, Hilden, Germany). 

RNA samples were quantified using a Qubit 4.0 Fluorometer (Life Technologies, Carlsbad, CA, USA), and RNA integrity was checked with an RNA kit on an Agilent 5300 Fragment Analyzer (Agilent Technologies, Palo Alto, CA, USA). RNA sequencing libraries were prepared using the NEBNext Ultra II RNA Library Prep Kit for Illumina following the manufacturer’s instructions (NEB, Ipswich, MA, USA). Briefly, mRNAs were first enriched with Oligo(dT) beads. Enriched mRNAs were fragmented according to the manufacturer’s instructions. First-strand and second-strand cDNAs were subsequently synthesized. cDNA fragments were end-repaired and adenylated at 3′ends, and universal adapters were ligated to cDNA fragments, followed by index addition and library enrichment by limited-cycle PCR. Sequencing libraries were validated using an NGS kit on the Agilent 5300 Fragment Analyzer (Agilent Technologies, Palo Alto, CA, USA) and quantified using the Qubit 4.0 Fluorometer (Invitrogen, Carlsbad, CA, USA). The sequencing libraries were multiplexed and loaded on the flow cell on the Illumina NovaSeq 6000 instrument according to the manufacturer’s instructions. The samples were sequenced using a 2 × 150 pair-end (PE) configuration in v1.5. Image analysis and base calling were conducted with NovaSeq Control software v1.7 on the NovaSeq instrument. Raw sequence data (.bcl files) generated from the Illumina NovaSeq were converted into fastq files and demultiplexed using the Illumina bcl2fastq program version 2.20. One mismatch was allowed for index sequence identification. Heatmap generation was achieved using the R package pHeatmap. The scale is relative gene expression, shown as rLog-transformed normalised counts.

### 2.4. GO and Pathway Enrichment Analysis

Gene ontology (GO) analysis was performed on the statistically significant set (padj < 0.05) of genes by implementing the software GeneSCF v.1.1-p2. The goa_human GO list was used to cluster the set of genes based on their biological processes and determine their statistical significance. Significantly, DEGs were clustered by their GO, and the enrichment of GO terms was tested using Fisher’s exact test (GeneSCF v1.1-p2). Up to 40 GO terms are significantly enriched with an adjusted *p*-value less than 0.05 in the DEG sets. Additionally, the Gene Ontology (GO) database (http://geneontology.org/, accessed on 5 August 2023) was employed in a separate analysis approach to characterise gene products based on their associated biological processes (BP), cellular compartments (CC) and molecular functions (MF) for the 599 differentially expressed genes (DEGs) with statistical significance (non-adj *p*-value less than 0.05) in the DEGs dataset (Table S1). The same approach was also used for the subsets of underrepresented and overrepresented DEGs, respectively. The top 10 GO terms from each category (BP, CC and MF) were extracted and incorporated in various enrichment plots (SRplot-Free online GO: http://bioinformatics.com.cn/en, accessed on 5 August 2023).

### 2.5. Reverse Transcription-Quantitative PCR (RT-qPCR)

RT-qPCR assays were performed as described previously [13,25] with minor modifications. Briefly, mRNA was purified directly from cells using the Dynabeads™ mRNA DIRECT™ Purification Kit (61012; Thermo Fisher Scientific, UK) and then used directly in the RT-qPCR reaction containing qPCRBIO SyGrene 1-Step Go Lo-ROX (PB25.31-12; PCRBiosystems, UK) and gene-specific primers for one-step reverse transcription and qPCR to quantify gene expression in the LightCycler 480 qPCR system (Roche, West Sussex, England, UK). Thermocycling began at 45 °C for 10 min (for reverse transcription) followed by 95 °C for 30 s prior to 45 cycles of amplification at 95 °C for 1 s, 60 °C for 1 s, 72 °C for 1 s and 78 °C for 1 s (data acquisition). A ‘touch-down’ annealing temperature intervention (66 °C starting temperature with a stepwise reduction of 0.6 °C/cycle; 8 cycles) was introduced before the amplification step to maximise primer specificity. Melting analysis (95 °C for 30 s, 75 °C for 30 s, 75–99 °C at a ramp rate of 0.57 °C/s) was performed at the end of qPCR amplification to validate single-product amplification in each well. Relative quantification of mRNA transcripts was calculated based on the second derivative maximum algorithm (Roche). Primer sequences are provided in Supplementary Table S2. All target genes were normalised to two stable reference genes. 

### 2.6. Immunofluorescence

Cells were seeded and grown on coverslips overnight before fixation in either 3.6% formaldehyde (for Cx43 staining, Sigma: C6219, rabbit anti-Cx43, gift from Diana Blaydon) in PBS or ice-cold methanol (for desmoplakin staining, mouse monoclonal 115F, gift from David Garrod) for 10 min. The samples fixed with formaldehyde were subsequently permeabilized with 0.1% Triton X-100 for 5 min. After blocking with 10% goat serum (Sigma) for 20 min, coverslips were incubated with the primary and secondary antibodies, respectively, each lasting for 1 h and washed 3 times at room temperature. Finally, coverslips were counterstained with DAPI for 8–10 min and were mounted on slides before image acquisition with a Leica DM5000 Epi-Fluorescence Microscope (Leica Microsystems (UK) Ltd., Milton Keynes, UK) or Zeiss 710 Laser Scanning Confocal Microscope (Carl Zeiss MicroImaging GmbH, Jena, Germany) (Blizard Institute Core Facility). Images were analysed with FIJI software (version 1.53). Student’s *t*-test was used to determine the *p*-values. *p* < 0.05 was considered statistically significant between the two group comparisons.

### 2.7. Western Blotting Analysis

This method was also described in our previous study [13]. Briefly, cells grown to confluence in a 6-well plate after 2 days were extracted with sodium dodecyl sulphate (SDS) Laemmli sample buffer (0.5 M Tris-Cl, pH 6.8, 4% SDS, 20% glycerol; 10% (*v*/*v*) 2-mercaptoethanol was added after protein assay). Protein concentration for each sample was determined by the DC Protein Assay (Bio-Rad, Hercules, CA, USA), and equal amounts of protein samples were loaded and resolved by SDS/PAGE and then transferred to a nitrocellulose membrane. After blocking the nonspecific binding sites on the membrane for 30 min in blocking buffer (5% *w*/*v* nonfat dry milk in TTBS containing 0.1% Tween 20), the membrane was incubated with primary antibody against specific proteins (Abcam: ab3208, mouse anti-MMP13 antibody (gift from Michael Allen); Sigma: C6219, rabbit anti-Cx43 and mouse anti-Cx43, clone 4E6.2, gifts from Diana Blaydon) at appropriate dilutions overnight at 4 °C. After three washes in TTBS, the membrane was incubated with the secondary antibody conjugated with HRP (rabbit-SAB3700846, mouse-A0168; Sigma) at appropriate dilutions in a blocking buffer for 1 h. After three washes, the membrane was subjected to ECL Western Blotting Substrate (Thermo Fisher Scientific). The membrane was then exposed to Amersham Hyperfilm ECL (VWR, Leicestershire, UK) and developed in an AGFA Curix 60 Developer (Blizard Institute Core Facility) in a dark room to detect target proteins.

### 2.8. Transmission Electron Microscopy (TEM)

The sample was embedded in Araldite resin (Agar Scientific Ltd., Essex, UK) and ultrathin sectioned for TEM analysis in JEM1400F (JEOL) at 120 kV. Images were taken with a Rio 16 CMOS camera (Gatan, AMETEK (GB) Limited, Leicester, UK).

## 3. Results

### 3.1. Differential Transcriptome Analysis in an Oral Squamous Carcinoma Cell Line with DSG3 Overexpression

Transcriptome profiles were compared between DSG3-overexpressing (referred to as ‘D’ hereafter; *n* = 2) and the matched empty vector control (referred to as ‘V’ hereafter; *n* = 2) cell lines derived from an oral SCC H413 line. A total of 72 million reads were generated. Following the extraction of gene hit counts, these counts were utilized to generate a table of gene hit counts. This table was subsequently employed for downstream differential expression analysis. Using DESeq2, a comparison of gene expression between the two sample conditions (D vs. V) was conducted. The Wald test was employed to generate *p*-values and log2 fold changes (Table S1). Genes with an adjusted *p*-value < 0.05 and an absolute log2 fold change > 1 were considered differentially expressed genes (DEGs) for this comparison (the cutoff value).

**Figure 1:**
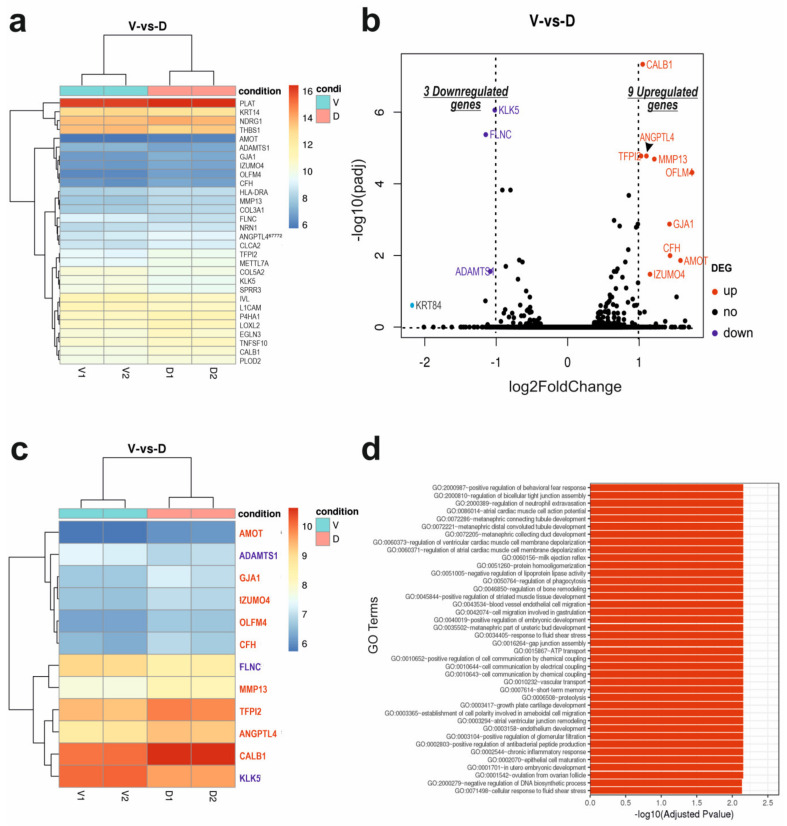
(**a**) A biclustering heatmap of the expression profile of the top 30 DEGs sorted by their adjusted *p*-value by plotting their log2 transformed expression values in four samples of the two conditions. (**b**) The volcano plot displays differentially expressed genes (DEGs) associated with DSG3. Here, the *x* axis represents the log2 fold change in each gene, while the *y* axis represents the log10 of its adjusted *p*-value. Each data point in the scatter plot represents a gene. Genes with an adjusted *p*-value less than 0.05 and a log2 fold change greater than 1 are marked as red dots, indicating upregulated genes. Blue dots represent downregulated genes, with an adjusted *p*-value less than 0.05 and a log2 fold change less than −1. The black dots represent the transcripts whose expression levels did not reach statistical significance between the two groups. (**c**) The biclustering heatmap of the expression profile of the 12 significant DEGs that are highlighted in B (also in Table S2). (**d**) Bar chart for the top 40 GO terms that are significantly enriched in biological processes with respect to the 12 significant DEGs based on the cutoff value. V: vector control group; D: Dsg3-overexpressing group.

First, a biclustering heatmap was constructed to visualise the expression profile of the top 30 genes sorted by their adjusted *p*-value by plotting their log2 transformed expression values of the two conditions in duplicate (Figure 1a). This analysis facilitates the identification of potential coregulated genes between two sample conditions. As shown in the heatmap, four genes such as PLAT, KRT14, NDRG1 and THBS1 are highly expressed, whereas another cluster of the genes including AMOT, ADAMTS1, GJA1, IZUMO4, etc., are expressed at low levels in both V and D cells. Other genes with intermediate mixed levels of expression were HLA-DRA, MMP-13, COL3A1, FLNC, KLK5, IVL and CALB1 genes, etc. (Figure 1a). It is worth noting that GJA1 is among the genes expressed at low levels, whereas MMP-13 is among those expressed at intermediate levels.

Next, a volcano plot was used to visualise the overall transcriptional changes and the number of significant DEGs between the two groups (Figure 1b and Table S1). Based on our stringent cutoff value (padj < 0.05 and an absolute log2 fold change > 1), overexpression of DSG3 led to a significant change in the expression of 12 genes, with the corresponding biclustering heatmap shown in Figure 1c (Table S3). Among these genes, nine (including GJA1, MMP-13, AMOT, CFH, OLFM4, IZUMO4, ANGPTL4, CALB1 and TFPI2) were upregulated, and three (ADAMTS1, FLNC and KLK5) were downregulated. However, based on the *p*-value (unadjusted) of less than 0.05 and an absolute log2 fold change > 1, 37 genes showed upregulation and 18 genes showed downregulation, including the 12 genes described above (Tables S4 and S5). 

### 3.2. Gene Ontology Enrichment and Pathway Analysis

Significant DEGs (shown in Figure 1b,c), including GJA1, MMP-13, OLFM4, ANGPTL4, ADAMTS1, AMOT and TFPI2, were clustered by their gene ontology (GO), and the enrichment of GO terms was tested using Fisher’s exact test (GeneSCF v1.1-p2). The results show that up to 40 GO terms are significantly enriched based on the cutoff value in the DEG sets (Figure 1d and Table S6). These genes are implicated in biological processes, including junction assembly (both the tight junctions and gap junctions) (GO:0016264; GO:2000810), cell communications, signal transduction, cell migration, epithelial cell differentiation and maturation (GO:0002070), development and cellular response to fluid shear stress, etc.

**Figure 2:**
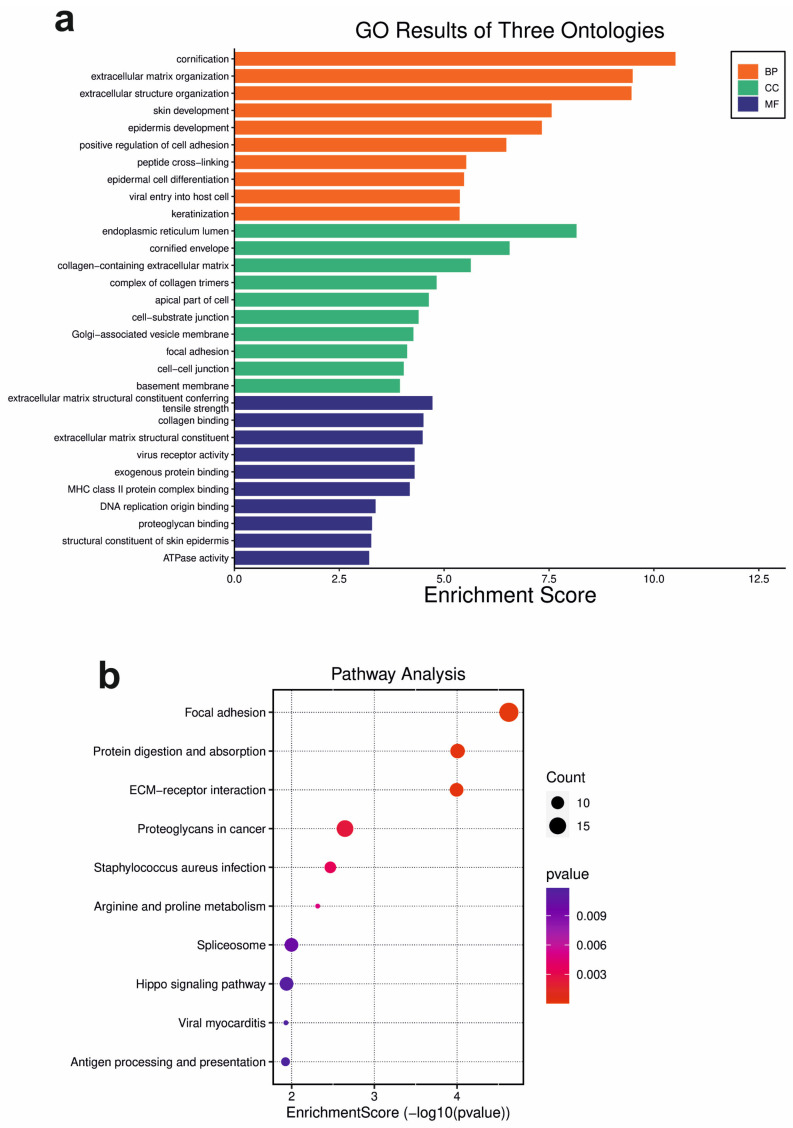
Gene ontology (GO) enrichment analysis of the DSG3 molecular network. (**a**) Bar chart for the significantly enriched gene ontology (GO) terms of differentially expressed genes (DEGs), with *p*-value < 0.05. BP: biological processes; CC: cellular components; MF: molecular function. The *y* axis shows GO terms, whereas the *x* axis denotes the enrichment score. (**b**) Bubble plot of pathway analysis. The size of each circle represents the number of genes. The colour scale indicates different thresholds of the *p*-value.

To gain a deeper understanding of the functional annotations and roles within the DSG3 molecular network, a comprehensive gene enrichment analysis was conducted. A total of 599 DEGs that display statistically significant changes in expression (*p* < 0.05) were analysed by employing robust and widely recognized resources—the Gene Ontology (GO) database (http://geneontology.org/, accessed on 5 August 2023) and SRPlot (https://www.bioinformatics.com.cn/en, accessed on 5 August 2023). The GO database provides meticulously curated and structured ontologies that characterise gene products based on their associated biological processes (BP), cellular compartments (CC) and molecular functions (MF). The top 10 GO terms with a high enrichment score from each category (BP, CC and MF) were extracted and integrated into various plots to visualise the functional enrichment results (SRPlot), as illustrated in Figure 2 of the presentation (Figures S1–S3).

Enrichment analysis of GO-BP revealed that gene products associated with DSG3 are primarily involved in several common functions, such as cornification, extracellular matrix (ECM) organisation and skin development (Figure 2a (orange bars) and Figure S1). Enrichment analysis of GO-CC showed that DSG3-associated gene products predominantly reside within a diverse array of structures, encompassing the endoplasmic reticulum lumen, cornified envelope and collagen trimer complex (Figure 2a (green bars) and Figure S2). In addition to cell–cell junction, cell–substrate junction and focal adhesion are also associated. Furthermore, enrichment analysis of GO-MF illuminates the versatile roles of DSG3-associated genes, including serving as structural components of the ECM (thus conferring tensile strength), serving as the binding of protein complexes (e.g., collagens, ECM structural constituent, exogenous proteins and major histocompatibility complex (MHC) protein complexes) and regulating viral receptor activity (Figure 2a (blue bars) and Figure S3). 

The pathway analysis (SRPlot) identified several important, previously unknown pathways concerning DSG3, including the focal adhesion pathway, protein digestion and absorption pathway, ECM-receptor interaction pathway and proteoglycan in cancer, etc. (Figure 2b). Interestingly, the Hippo signalling pathway, which we previously reported to have an association with DSG3 [13], is also listed within the top 10 enrichment pathways.

**Figure 3:**
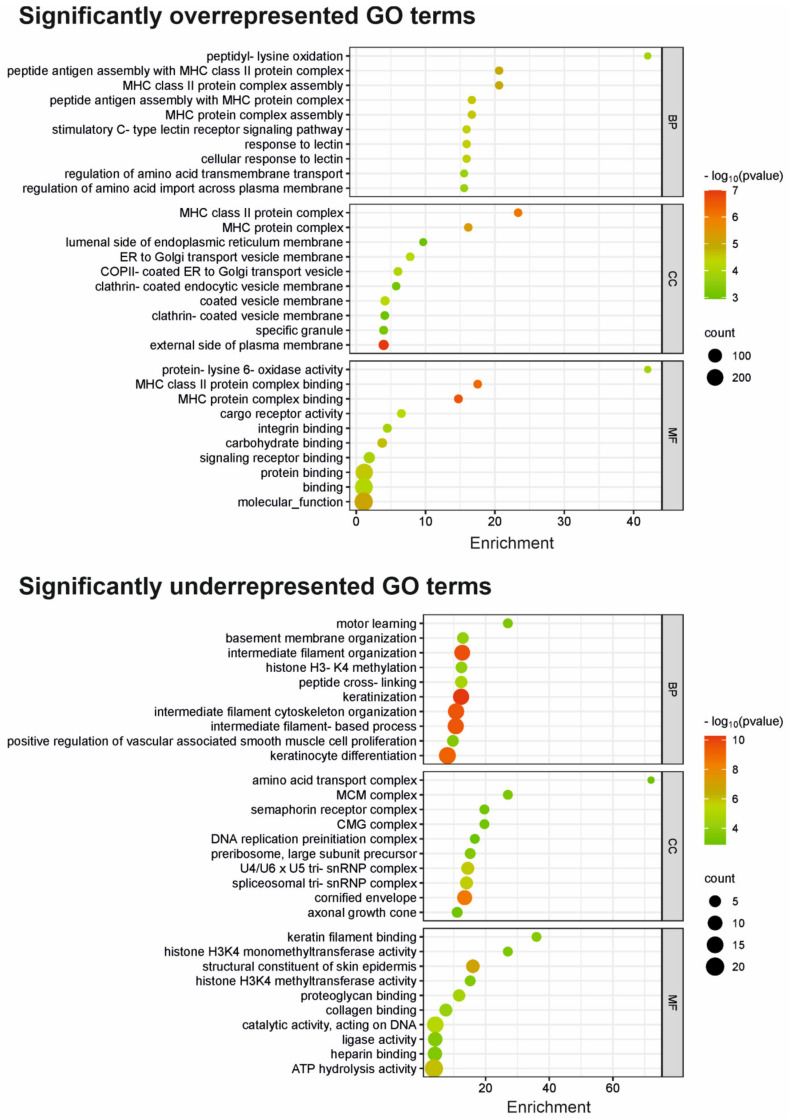
Gene ontology (GO) based on over- and underrepresentation analysis of the DSG3 molecular network. The bubble plots show overrepresentation analysis results (**top panel**) and underrepresentation analysis results (**bottom panel**) of the DSG3-associated genes. The *y* axis shows the top 10 significantly (*p* < 0.05) enriched GO terms, and the *x* axis displays the enrichment score. BP: biological processes; CC: cellular components; MF: molecular function. The colour scale indicates different thresholds of the *p*-value. The numbers of candidate genes annotated with a GO term are mapped to the scatter plot by point size.

Moreover, GO enrichment analysis was performed on a subset of overrepresented (313) and underrepresented (286) genes, respectively, and revealed interesting insights into the biological processes and functions in which these genes are involved (Figure 3). Among the overrepresented genes, peptidyl lysine oxidation (GO:0018057) is significantly enriched in the biological process and is involved in collagen synthesis or modification. Other DSG3-associated overrepresented genes are involved in the assembly and binding of various protein complexes, including MHC complexes, cargo receptors and integrins. This indicates their importance in processes like immune response, cellular transport and cell adhesion. C-type lectin receptor signalling pathway (GO:0002223), among the overrepresented biological processes, suggests an involvement of these genes in immune response and signal transduction through C-type lectin receptors. On the other hand, the underrepresented genes are enriched for amino acid transport complex (GO:1990184). Other underrepresented genes are associated with intermediate filament organisation, keratinocyte differentiation and cornified envelope, which are important for maintaining cell structure and integrity, keratinocyte maturation and differentiation and skin barrier function. 

Overall, the GO term enrichment analysis collectively provides valuable insights into the roles and functions of DSG3 and its associated gene products in a cancer cell background. It highlights the involvement of DSG3 in processes related to cell adhesion, extracellular matrix dynamics, skin development and potential contributions to digestive and absorption mechanisms. Although these findings are from a cancer cell line, they may imply a noncancerous background. Together, these analyses provide valuable context for understanding the broader biological significance of DSG3 in various cellular and physiological processes.

### 3.3. Validation of Gene Expression by Quantitative Real-Time PCR (qPCR)

**Figure 4:**
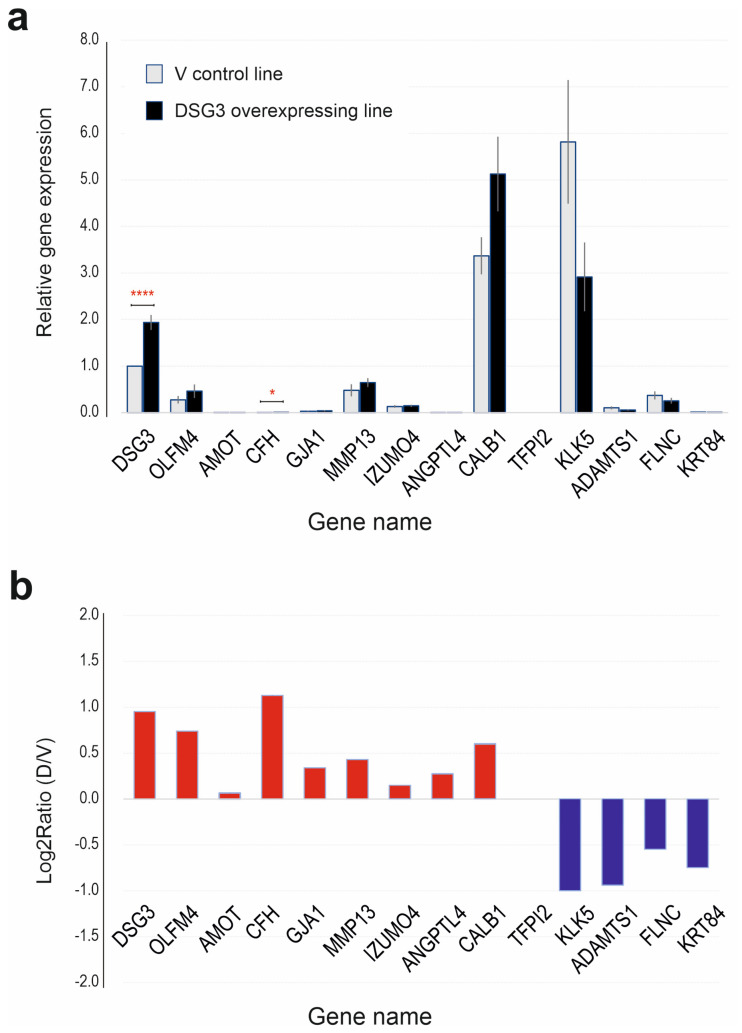
Validation of gene expression by RT-qPCR. (**a**) qPCR data indicating the relative gene expression (*n* = 6, pooled from three independent biological samples, mean ± SEM, * *p* < 0.05, **** *p* < 0.0001 determined by Student’s *t*-test). (**b**) Log2 ratio of D (DSG3-overexpressing) over V (vector) control samples. Except for TFPI2, which was undetectable, all other genes showed a similar trend to that of the RNA-Seq data (Figure 1).

To confirm the RNA-Seq results of the DEGs with adjusted *p*-values less than 0.05 (shown in Figure 1a,c), RT-qPCR was performed to validate all those listed significant DEGs, including both the upregulated and downregulated genes, with GAPDH and POLR2A as the reference genes (Table S2). Cells were grown in the same way as those prepared for RNA-Seq. Three biological replicates for each group with two repetitions for each run of qPCR were analysed, and the resulting data are presented in Figure 4. As indicated, DSG3 had a nearly 2-fold increase in DSG3-overexpressing cells compared to the empty vector control. However, we found that TFPI2 was undetectable. A few other genes such as AMOT, CFH, GJA1 and ANGPTL4 were expressed at extremely low levels (Figure 4a); this finding is consistent with RNA-Seq (Figure 1a). Overall, the differential gene expression patterns of the panel of selected genes measured by RT-qPCR (Figure 4b) correlated with that of RNA-Seq results (Figure 1b,c). Additionally, we included the KRT84 gene in this analysis, as this gene has been reported as a potential tumour suppressor in oral SCC, with the expression level showing a decreasing tendency along with an increase in tumour grade [26]. While RNA-Seq found a 4.5-fold reduction in KRT84 in D cells compared to V control (*p* < 0.001), RT-qPCR only detected a ~0.4-fold difference due to its low expression level in the H413 line.

### 3.4. Validation of Gene Expression by Western Blotting and Immunofluorescence Analyses

**Figure 5:**
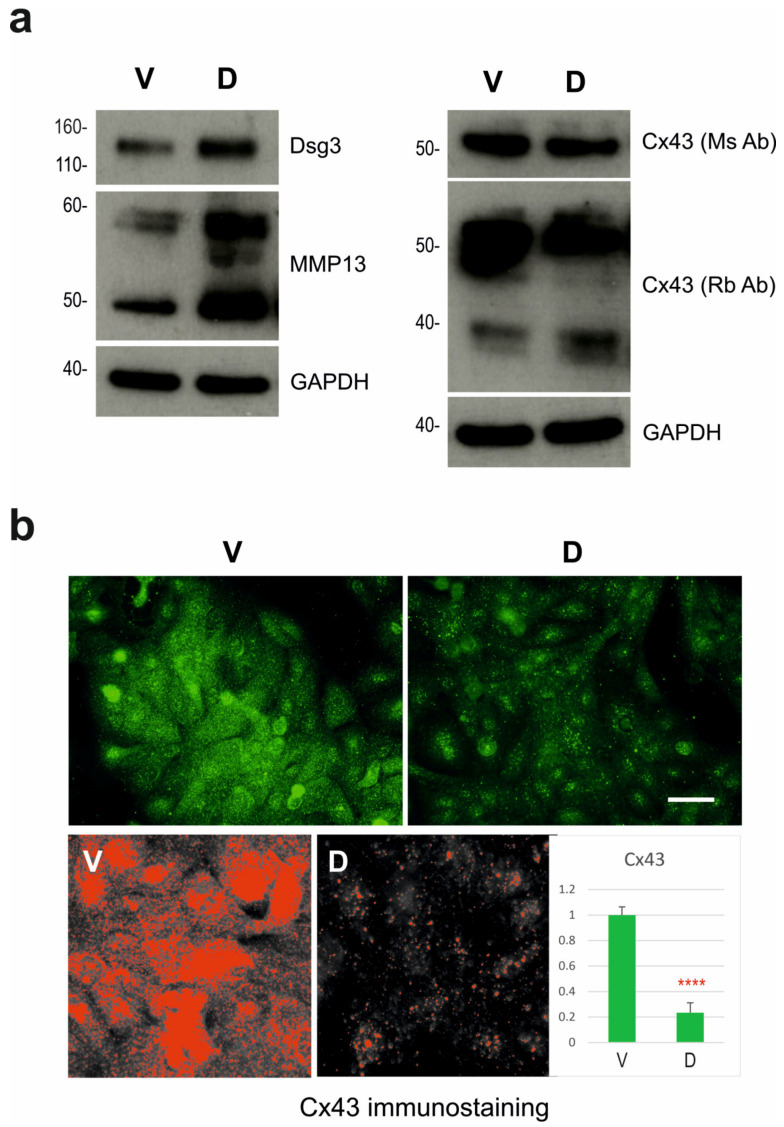
Validation of the protein expression for MMP-13 and connexin-43 (Cx43). (**a**) Western blotting analysis of the indicated proteins. GAPDH was used as a loading control. While MMP-13 showed an increase in the DSG3-overexpressing cells (D) compared to V (vector) control, Cx43 exhibited an opposite trend with a reduction in the D cell line. Two antibodies for Cx43 raised in two different species were tested here. (**b**) Cx43 immunostaining in V control and D cells (green). Below is the image quantitation highlighting the pixels above a threshold. The quantitation data are displayed in the bar chart on the right (mean ± SD, **** *p* < 0.0001).

To validate the DEG findings at the protein level, Western blotting and fluorescent analyses were employed. Here, we focused on two genes, GJA1 and MMP-13, which are related to junction assembly and the breakdown of the ECM, respectively. MMP-13, also known as collagenase 3, is the key enzyme in the cleavage of type II collagen and plays a pivotal role in ECM remodelling [27]. GJA1 encodes the gap junction alpha-1 protein, also known as connexin 43 (Cx43), and is a component of gap junctions which allow for gap junction communication between cells to regulate cell death, proliferation and differentiation [28]. First, the protein expression of DSG3 in two conditions was verified, which demonstrated an elevated level of DSG3 expression in D (DSG3-overexpressing) cells relative to V (vector) control (Figure 5a (left)). In line with the RNA-Seq and RT-qPCR data, the expression of MMP-13 showed an evident increase in D cells compared to the control. MMP-13 contains two forms, i.e., pro-collagenase-3 (Mr 60,000) to the fully active enzyme (Mr 48,000) [29]. We showed that DSG3 overexpression induces expression of both pro and fully activated forms) of MMP-13. However, unexpectedly, we found a decrease in Cx43 using Western blotting analysis, rather than an increase as indicated by RNA-Seq, in D cells, and this was confirmed using two different specific Cx43 antibodies raised in different species, i.e., mouse monoclonal and rabbit polyclonal against Cx43, both of which showed a similar reduction in Cx43 (Figure 5a (right)). In addition, rabbit anti-Cx43 antibody also detected a lower band of approximately 38 kDa that displayed a moderate increase in D cells relative to the control sample. Furthermore, immunofluorescent staining with the rabbit anti-Cx43 antibody indicated a significant reduction in D compared to V control cells (*p* < 0.001, Figure 5b). The distribution of Cx43 in H413 cells was predominantly located in the cytoplasm and nucleus with little presentation at the cell periphery. These results collectively indicated a consistent finding for MMP-13 but an opposite trend for Cx43 compared to the results obtained by RNA-Seq and qRT-PCR analyses despite the fact that both techniques detected low mRNA expression levels of GJA1 in the H413 cell lines. 

### 3.5. Desmosome Morphology by Transmission Electron Microscopy

**Figure 6:**
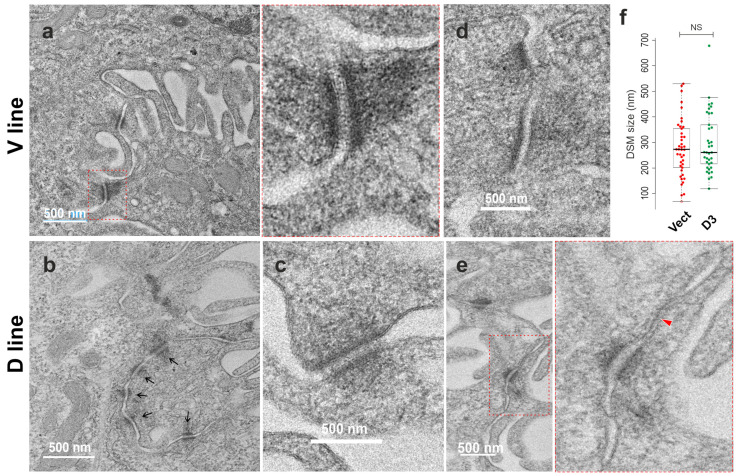
Transmission electron microscopy (TEM) of H413-D3 (DSG3-overexpressing) and H413-V control lines. Cells were grown to confluence for two days and then were treated with dispase (2.4 U/mL) for 20 min to detach the epithelial sheets. The samples were fixed immediately with glutaraldehyde overnight and stored in a fridge prior to TEM processing. (**a**–**c**) showed characteristic desmosomes with symmetrical electron-dense structures in both V control and D samples. However, those presented in D cells appeared slightly less electron-dense than those in V control cells. A cluster of desmosomes was captured in the D sample (black arrows). (**d**) displays two half-desmosomes presented only in one of the adjacent cells. (**e**) shows an immature desmosome adjacent to a short stretch of gap junction (red arrow). The enlarged images for the red dotted line boxes are displayed on the right. (**f**) shows a box whiskers plot of the desmosome size, which was measured in two conditions (V: *n* = 42; D: *n* = 37, Midline: median). No statistical significance (NS) was detected between them. Scale bars are 500 nm.

**Figure 7:**
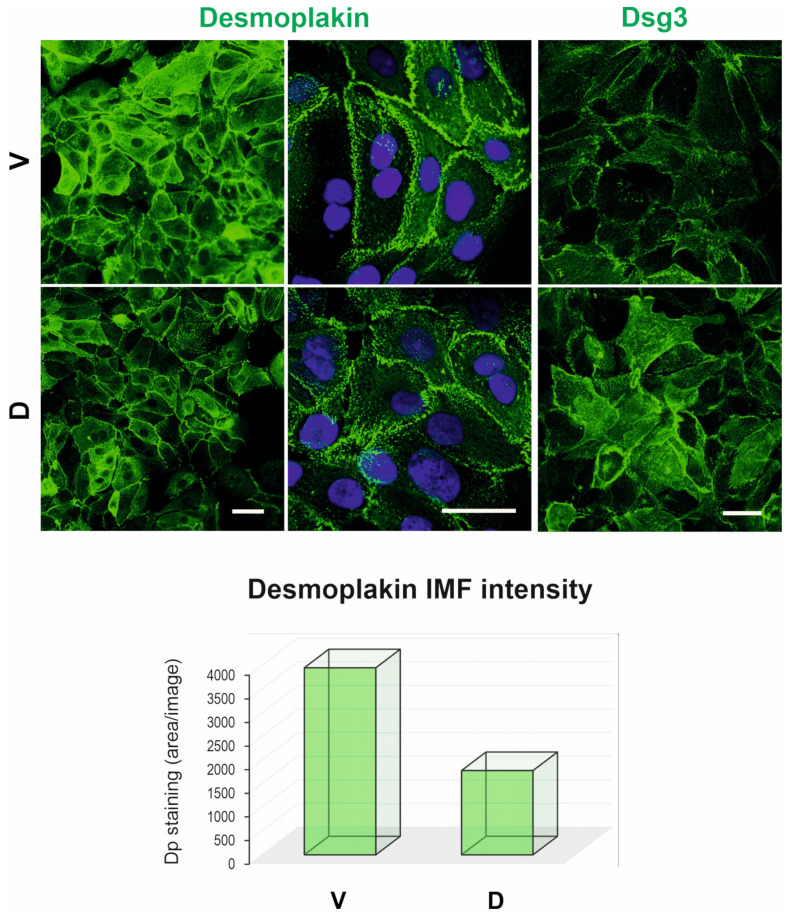
Confocal images for desmoplakin and DSG3 staining, respectively. Both proteins are predominantly distributed at the cell borders but with more enhanced and diffuse DSG3 cytoplasmic staining in D cells compared to V control. The quantitation data are shown underneath the images. Scale bars are 20 µm.

The discrepancy in GJA1/Cx43 expression by RNA-Seq and protein analyses prompted us to explore the morphology of cell–cell junctions in the V and D cell lines. To this end, traditional transmission electron microscopy (TEM) was performed to investigate in-depth the desmosome morphology and structures. Cells were seeded at confluent density and grown for 2 days to allow the formation of epithelial monolayers. Subsequently, the epithelial sheets were detached using dispase (2.4 U/mL) treatment [13] and fixed for TEM examinations. Close inspection found desmosomes with characteristic symmetrical electron-dense structures sandwiching the extracellular desmoglea core domain, which are sparsely located at the lateral cell borders in both samples (Figure 6). They were typically found between the membrane projections of neighbouring cells. Some of them showed a clear connection with the intermediate filaments at the inner dense plaques (Figure 6a–c). Interestingly, in a region of the D cell sample, a cluster of five desmosomes with varied electron densities, sizes and degrees of connection with the intermediate filaments was found (arrows Figure 6b). Occasionally, half-desmosomes were visible, lining the plasma membrane of one of the neighbouring cells (Figure 6d). However, it appeared that no apparent difference in the desmosome frequency was observable between the two conditions. In general, the desmosomes in D cells showed as less electron-dense compared to the V control sample, suggesting less connection with keratin intermediate filaments and/or less maturity in desmosome formation. Notably, a short stretch of gap junction located adjacent to an immature desmosome was captured in the D sample (red arrowhead in the insert Figure 6e). The desmosome size was measured with ImageJ (V: *n* = 42; D: *n* = 37), and the result showed no statistically significant difference between the two conditions (Figure 6f). In general, the average size of desmosomes in H413 cells measured slightly less than 300 nm (median ± SD: V, 272.24 ± 113.04; D, 260.83 ± 116.98). To complement these findings, we performed immunofluorescent staining for desmoplakin (Dp), the marker of desmosomes. Confocal microscopy showed a typical punctate Dp staining pattern with predominant periphery distribution. However, slightly fewer fluorescent signals were shown in D cells than in V control (Figure 7), suggesting less connection with keratin intermediate filaments. Taken together, these findings indicate that overexpression of DSG3 seemed not to promote the desmosome maturation; the results are consistent with the notion that DSG3 overexpression does not significantly enhance cell–cell adhesion in oral SCC H413 cells [13].

## 4. Discussion

DSG3 exhibits increased expression in SCC across various organ systems, yet a complete understanding of its intricate biological effects remains unexplored. Our prior in vitro study found that heightened DSG3 expression in oral SCC H413 cells restrains synchronized cell migration by inducing YAP phosphorylation and subsequently attenuating its transcriptional activity in the nucleus, and this is achieved via inhibiting EGFR signalling [13]. RNA-Seq, a comprehensive technique capturing the entire transcriptome, offers insights into gene expression, functional diversity and disease mechanisms. This current study aimed to expand our previous report [13] to uncover differentially expressed genes (DEGs) between DSG3-overexpressing and vector control-expressing oral SCC cells (H413) in order to broaden our understanding of DSG3 in oral cancer cells. The analysis identified 599 DEGs, with 286 genes downregulated and 313 upregulated, which are associated with DSG3 expression. These DEGs are involved in various biological processes, including collagen formation, cell adhesion, immune response, amino acid transport and keratinocyte function. 

This study unveils DSG3′s pivotal role in critical cellular processes like cell adhesion, collagen formation and ECM organisation, primarily through the integrin-mediated focal adhesion pathway. We confirm DSG3′s active promotion of MMP-13 expression, an enzyme that degrades ECM components, including key collagens found in skin, tendons and cartilage, crucial for tissue integrity. This physiological function is essential for processes such as wound healing, embryogenesis and tissue homeostasis. However, when MMPs become dysregulated or overactive such as in cancer, MMPs can transcend normal physiology, influencing cancer progression. Heightened MMP-13 expression, especially in cancer, facilitates tumour invasion into nearby tissues. MMP-13′s enzymatic activity reshapes the tumour microenvironment, favouring tumour advancement. Elevated MMP-13 in various cancers is linked to increased invasiveness, metastasis and poor prognosis [29]. Thus, targeting MMP-13 holds promise for anticancer therapies. Interestingly, DSG3-overexpressing cells display enriched peptidyl lysine oxidation (GO:0018057), vital for collagen synthesis and tissue strength. This process hydroxylates lysine in procollagen, forming cross-links that bolster collagen-rich tissues. It is worth noting that the connective tissue in the oral mucous membrane is referred to as the lamina propria, characterised by its loose connective tissue composition with fewer collagen fibres. In the context of oral cancer, DSG3 is likely involved in modifying the tumour microenvironment by promoting collagen deposition, thereby facilitating tumour progression. Moreover, this study highlights integrins, essential signalling receptors, in the DSG3 context. Integrins drive intracellular cascades upon ligand binding, supporting adhesion, migration and other vital cellular processes. Taken together, the increase in collagen, integrin support and MMP activity can facilitate ECM remodelling and promote the invasion of cancer cells into surrounding tissues that potentially contribute to the metastatic progression of oral cancer. Unravelling DSG3′s impact on focal adhesion and integrin complex assembly awaits further exploration in future research.

GJA1, a member of the highly conserved connexin family, encodes the protein connexin-43 (Cx43). Connexins form hexameric complexes called connexons on the cell membrane, acting as hemichannels for direct communication and ion/molecule exchange between neighbouring cells. This gap junction function is crucial for various physiological processes, including skin development, cornified envelope formation related to skin barrier function and overall skin homeostasis. Disruptions in gap junctions are implicated in diverse diseases, including cancers [30]. Cx43 notably facilitates gap junction-mediated intercellular communication in the skin, contributing to processes like epidermal maturation (GO:0002070~epithelial cell maturation). Applying rigorous criteria (padj < 0.05 and log2 fold change >1), we identified, for the first time, GJA1 as one of the nine upregulated DEGs in the context of DSG3 influence (GO:0016264~gap junction assembly). As confirmed by both RNA-Seq and RT-qPCR techniques, GJA1 mRNA appeared to be expressed at low levels in the H413 cell line. Nevertheless, the gene product of GJA1 (Cx43 protein) was readily detectable by Western blotting and immunofluorescence. In contrast to RNA-Seq and qPCR, which showed a Cx43 increase in cells with DSG3 overexpression, Western blot analysis revealed a modest decrease in Cx43 protein levels in DSG3-overexpressing cells compared to the control, and this result was further corroborated by immunostaining and quantification of CX43 expression. In general, this finding aligns with the idea that connexins act as cancer suppressors [30], and the increased level of DSG3 further contributes to the reduction in Cx43 expression. Intriguingly, Cx43 distribution in H413 cells appeared concentrated in the cytoplasm and nucleus rather than the cell periphery, suggesting a potential irregular gap junction function or enhanced protein turnover or the specific gap junction-independent mechanism operating in this oral cancer cell line. This phenomenon awaits further exploration [30]. 

While DSG3 is known to enhance junction assembly as expected, analysis of biological processes and functions reveals that DSG3 appears to inhibit intermediate filament organisation (GO:0045109; GO:0045104; GO:0045103), keratinocyte differentiation (GO:0030216; GO:0009913), cornified envelope formation (GO:0001533), skin development (GO:0031424; GO:0043588) and hair cycle (GO:0042633). Such inhibitions are associated with the downregulation of KLK5 (kallikrein-related peptidase 5) and other differentiation markers (such as IVL) linked to DSG3 expression. KLK5 plays a role in processing profilaggrin, contributing to keratinocyte differentiation and the functional skin barrier. All these concepts listed here are interconnected, collectively influencing keratinocyte differentiation and the protective skin barrier. Skin development is intricate, involving multiple layers, cell types and structures. The cornified envelope, formed during late keratinocyte differentiation, serves as a vital shield against water loss and pathogens, contributing to overall skin barrier function. Intermediate filaments are part of the cytoskeleton, providing structural support for keratinocytes and maintaining epidermal integrity. Notably, in line with these findings, our previous research indicated that DSG3 overexpression does not evoke significant enhancement in cell–cell adhesion strength in H413 cells compared to controls (i.e., no difference in cell–cell adhesion between the two conditions assessed by dispase-based cell dissociation assay) [13], suggesting that DSG3 may not necessarily be essential for junction adhesion and stability. To further our knowledge, here we conducted transmission electron microscopy to investigate the morphology of intercellular junctions, in particular gap junctions and desmosomes. While the characteristic gap junctions were scarce in both samples, scattered desmosomes were detectable along plasma membrane projections. We noticed no apparent increase in desmosome frequency in DSG3-overexpressing cells, though occasional clusters were observed, with lower electron density than control samples. Nevertheless, although the desmosomes appeared slightly smaller in DSG3-overexpressing cells, there was no indication of statistical significance for desmosome size between the two conditions. Consistent with the downregulation of KRT14 (keratin 14), immunostaining for Dp, which anchors keratin intermediate filaments to desmosomes, showed reduced Dp expression in cells with DSG3 overexpression compared to vector control cells, suggesting less mature desmosomes in DSG3-overexpressing cells than control samples. Collectively, integrating RNA-Seq, bioinformatics and laboratory experimental work prompted us to propose that while DSG3 facilitates junction assembly, it may not be necessarily essential for junction maturation. In other words, DSG3 is involved in the early stages of creating desmosomal junctions, but as these junctions become more established, the role of DSG3 might become less critical. Other proteins/processes may take over to complete desmosome maturation. Alternatively, these results may simply reflect the more dynamic nature or dysregulation of the desmosomes in oral cancer cells with overexpression of DSG3. 

This study identifies changes in several genes whose roles in cancer are controversial. It shows an association between DSG3 expression and AMOT, a component of the Hippo network. While AMOT family members typically promote cancer cell proliferation and invasion in most cancers, they have opposing effects in lung cancer [31]. The regulation of YAP by AMOT remains unclear, with controversies in the literature [31]. Other DSG3-associated cancer-related genes include upregulated OLFM4 (antiapoptotic factor), downregulated ADAMTS1 (antiangiogenic activity), downregulated KRT84 (cancer suppressor in oral SCC) [26] and FLNC [32]. OLFM4, olfactomedin-4, is reported to be significantly increased in head and neck squamous cell carcinoma (75% tested), suggesting a potential biomarker in this type of cancer [33]. ADAMTS1 can have either a protumourigenic or antitumour progressive role depending on the cell context [32]. KRT84 has been implicated as a tumour suppressor and a good prognostic indicator for oral SCC. Low KRT84 expression level is shown to have inferior overall survival independent of multiple factors [26]. Overexpression of DSG3 caused a further reduction in KRT84. Filamin (FLNC), an actin-binding protein of the filamin family and anchoring several transmembrane proteins to the actin cytoskeleton, serves diverse structural and signalling roles [34]. It plays contrasting roles in contractile myocytes and noncontractile cancer cells. In contractile myocytes, FLNC is linked with Hippo, regulating migration and differentiation. Conversely, in cancer, FLNC promotes cell proliferation and migration [35]. In general, these findings suggest a potential oncogenic role of DSG3 in oral SCC cells, as proposed previously [2,36,37]. Another interesting finding from this study is that DSG3 plays a significant role in MHC complex assembly and binding (GO:0002399, GO:0002501, GO:0002396, GO:0042613, GO:0042611), pointing to its involvement in immune regulation, as demonstrated in pemphigus autoimmune blistering disease [38,39,40]. 

Characterised as a member of the cadherin cell adhesion molecule superfamily, DSG3 is primarily responsible for mediating intercellular adhesion. It is enriched in the basal layer of the epidermis but is uniformly expressed in the entire stratified squamous epithelial membrane of the oral mucosa. Squamous cell carcinoma originates from squamous epithelial cells, where cell–cell junctions are crucial not only for adhesion but also for signalling, in regulating cellular functions such as cell proliferation, differentiation and survival. Not all cancer cells undergo epithelial-to-mesenchymal transition (EMT), and many squamous carcinoma cells still retain their epithelial characteristics. Often, carcinoma cells undergo collective invasion while maintaining some cell–cell adhesion. Therefore, adhesion molecules like DSG3 may be vital in cancer cell adhesion, signalling and survival. In support of this notion, our previous studies suggest that DSG3 acts as an antistress, prosurvival protein in epithelial cells via suppression of p53 and reactive oxygen species [41,42,43]. Moreover, a recent comprehensive study based on 15,869 tissue microarrays identified that DSG3 expression is predominant in squamous cell carcinomas [20]. Loss of adhesion molecules such as DSG3 as well as E-cadherin occurs when cancer cells undergo dedifferentiation or EMT.

Moreover, we invested further in the DEGs from our study on head and neck cancer (HNSC) tumour samples compared with normal mucosa using the GEPIA online database tool (http://gepia.cancer-pku.cn/detail.php?clicktag=degenes, accessed on 14 November 2023). GEPIA is a newly developed interactive web server for analysing the RNA-Seq data of 9736 tumours and 8587 normal samples from the TCGA and the GTEx projects. In agreement with a potential oncogenic role for DSG3, the two key DEGs that we focused on in this study (GJA1 and MMP13) are both significantly upregulated in primary HNSC. Furthermore, of the four downregulated genes found in our study, two genes (FLNC and KRT84) showed a downregulated trend in HNSC, although these were not significant.

## 5. Conclusions

This research sheds light on the complex molecular landscape orchestrated by DSG3 in oral SCC cells, with some functions likely having potential applications in noncancerous backgrounds, e.g., normal keratinocytes. This study uncovers its pivotal roles in influencing critical processes such as cell adhesion, ECM organisation, junction assembly and the regulation of genes linked to cancer progression and skin development. However, some processes, such as intermediate filament organisation and keratinocyte differentiation, appear to be suppressed by DSG3 overexpression, highlighting the intricate and multifaceted effects of DSG3 in oral squamous carcinoma or keratinocytes in general. Further investigation is needed in order to fully understand the precise mechanisms and potential therapeutic implications of these findings. Overall, this study employed a robust approach to explore the functional annotation of DSG3-associated genes and to comprehend their molecular networks in oral cancer cells. As with any bioinformatics study, there exist certain limitations. This study was conducted using four libraries from two matched cell lines in H413, and it is important to note that not all functional aspects are fully represented by sequences alone, meaning that the genetic or molecular sequence of a biological molecule such as DNA or RNA or protein does not provide a complete understanding of its function or behaviour. Furthermore, due to the nature of cancer heterogeneity, other cancer cell lines may present varied landscapes. Of course, a larger sample size (e.g., three samples in each condition) in various cell lines would be helpful to provide more precise predictions of biological processes. Although the attempts at experimental work were undertaken to confirm some findings, further validation studies, especially in oral cancer patients, are warranted to support the conclusions drawn from this study. Nevertheless, as suggested in a recent report [44], it is likely that DSG3 is not required for tumour initiation but plays an important role in metastasis. 

## Data Availability

The data presented in this study are available on request from the corresponding author.

## Supplementary Materials

The following are available online at https://www.mdpi.com/article/10.3390/cells12232710/s1, Figure S1. Visualisations of gene ontology (GO) enrichment analysis of biological processes for differentially expressed genes (DEGs) with *p*-values less than 0.05; Figure S2. Visualisations of gene ontology (GO) enrichment analysis of cellular components for differentially expressed genes (DEGs) with *p*-values less than 0.05; Figure S3. Visualisations of gene ontology (GO) enrichment analysis of molecular functions for differentially expressed genes (DEGs) with *p*-values less than 0.05; Table S1. DEG table, Table S2. Primers; Table S3. Significantly differentially expressed genes; Table S4. Thirty−seven upregulated genes; Table S5. Eighteen downregulated genes; Table S6. GO_analysis.

## References

[1] Chen Y.J., Lee L.Y., Chao Y.K., Chang J.T., Lu Y.C., Li H.F., Chiu C.C., Li Y.C., Li Y.L., Chiou J.F. (2013). DSG3 facilitates cancer cell growth and invasion through the DSG3-plakoglobin-TCF/LEF-Myc/cyclin D1/MMP signaling pathway. PLoS ONE.

[2] Brown L., Wan H. (2015). Desmoglein 3: A help or a hindrance in cancer progression?. Cancers.

[3] Cheng J., Yang J., Xue K., Zhao Y., Zhao C., Li S., Wang Z. (2019). Desmoglein 3 Silencing Inhibits Inflammation and Goblet Cell Mucin Secretion in a Mouse Model of Chronic Rhinosinusitis via Disruption of the Wnt/beta-Catenin Signaling Pathway. Inflammation.

[4] Uraguchi M., Morikawa M., Shirakawa M., Sanada K., Imai K. (2004). Activation of WNT family expression and signaling in squamous cell carcinomas of the oral cavity. J. Dent. Res..

[5] Tsang S.M., Liu L., The M.T., Wheeler A., Grose R., Hart I.R., Garrod D.R., Fortune F., Wan H. (2010). Desmoglein 3, via an interaction with E-cadherin, is associated with activation of Src. PLoS ONE.

[6] Tsang S.M., Brown L., Lin K., Liu L., Piper K., O’Toole E.A., Grose R., Hart I.R., Garrod D.R., Fortune F. (2012). Non-junctional human desmoglein 3 acts as an upstream regulator of Src in E-cadherin adhesion, a pathway possibly involved in the pathogenesis of pemphigus vulgaris. J. Pathol..

[7] Rötzer V., Hartlieb E., Vielmuth F., Gliem M., Spindler V., Waschke J. (2015). E-cadherin and Src associate with extradesmosomal Dsg3 and modulate desmosome assembly and adhesion. Cell Mol. Life Sci..

[8] Marchenko S., Chernyavsky A.I., Arredondo J., Gindi V., Grando S.A. (2010). Antimitochondrial autoantibodies in pemphigus vulgaris: A missing link in disease pathophysiology. J. Biol. Chem..

[9] Bektas M., Jolly P.S., Berkowitz P., Amagai M., Rubenstein D.S. (2013). A pathophysiologic role for epidermal growth factor receptor in pemphigus acantholysis. J. Biol. Chem..

[10] Minabe M., Akiyama Y., Higa K., Tachikawa T., Takahashi S., Nomura T., Kouno M. (2019). A potential link between desmoglein 3 and epidermal growth factor receptor in oral squamous cell carcinoma and its effect on cetuximab treatment efficacy. Exp. Dermatol..

[11] Li X., Ishii N., Ohata C., Furumura M., Hashimoto T. (2014). Signalling pathways in pemphigus vulgaris. Exp. Dermatol..

[12] Ri H., Peiyan Z., Jianqi W., Yunteng Z., Gang L., Baoqing S. (2019). Desmoglein 3 gene mediates epidermal growth factor/epidermal growth factor receptor signaling pathway involved in inflammatory response and immune function of anaphylactic rhinitis. Biomed. Pharmacother..

[13] Ahmad U.S., Parkinson E.K., Wan H. (2022). Desmoglein-3 induces YAP phosphorylation and inactivation during collective migration of oral carcinoma cells. Mol. Oncol..

[14] Abula Y., Su Y., Tuniyazi D., Yi C. (2021). Desmoglein 3 contributes to tumorigenicity of pancreatic ductal adenocarcinoma through activating Src-FAK signaling. Anim. Cells Syst..

[15] Schmitt T., Hudemann C., Moztarzadeh S., Hertl M., Tikkanen R., Waschke J. (2023). Dsg3 epitope-specific signalling in pemphigus. Front. Immunol..

[16] Amber K.T., Valdebran M., Grando S.A. (2018). Paraneoplastic autoimmune multiorgan syndrome (PAMS): Beyond the single phenotype of paraneoplastic pemphigus. Autoimmun. Rev..

[17] Hakuno M., Shimizu H., Akiyama M., Amagai M., Wahl J.K., Wheelock M.J., Nishikawa T. (2000). Dissociation of intra- and extracellular domains of desmosomal cadherins and E-cadherin in Hailey-Hailey disease and Darier’s disease. Br. J. Dermatol..

[18] Hu Z., Bonifas J.M., Beech J., Bench G., Shigihara T., Ogawa H., Ikeda S., Mauro T., Epstein E.H. (2000). Mutations in ATP2C1, encoding a calcium pump, cause Hailey-Hailey disease. Nat. Genet..

[19] Patel V., Martin D., Malhotra R., Marsh C.A., Doci C.L., Veenstra T.D., Nathan C.A., Sinha U.K., Singh B., Molinolo A.A. (2013). DSG3 as a biomarker for the ultrasensitive detection of occult lymph node metastasis in oral cancer using nanostructured immunoarrays. Oral Oncol..

[20] Viehweger F., Azem A., Gorbokon N., Uhlig R., Lennartz M., Rico S.D., Kind S., Reiswich V., Kluth M., Hube-Magg C. (2022). Desmoglein 3 (Dsg3) expression in cancer: A tissue microarray study on 15,869 tumors. Pathol. Res. Pract..

[21] Teh M.T., Parkinson E.K., Thurlow J.K., Liu F., Fortune F., Wan H. (2011). A molecular study of desmosomes identifies a desmoglein isoform switch in head and neck squamous cell carcinoma. J. Oral Pathol. Med..

[22] Hiraki A., Shinohara M., Ikebe T., Nakamura S., Kurahara S., Garrod D.R. (1996). Immunohistochemical staining of desmosomal components in oral squamous cell carcinomas and its association with tumour behaviour. Br. J. Cancer.

[23] Depondt J., Shabana A.H., Florescu-Zorila S., Gehanno P., Forest N. (1999). Down-regulation of desmosomal molecules in oral and pharyngeal squamous cell carcinomas as a marker for tumour growth and distant metastasis. Eur. J. Oral Sci..

[24] Prime S.S., Eveson J.W., Stone A.M., Huntley S.P., Davies M., Paterson I.C., Robinson C.M. (2004). Metastatic dissemination of human malignant oral keratinocyte cell lines following orthotopic transplantation reflects response to TGF-beta 1. J. Pathol..

[25] Teh M.T., Hutchison I.L., Costea D.E., Neppelberg E., Liavaag P.G., Purdie K., Harwood C., Wan H., Odell E.W., Hackshaw A. (2013). Exploiting FOXM1-orchestrated molecular network for early squamous cell carcinoma diagnosis and prognosis. Int. J. Cancer.

[26] Liu Y., Li R., Ren G. (2020). KRT84 is a potential tumor suppressor and good prognosis signature of oral squamous cell carcinoma. Biosci. Rep..

[27] Leeman M.F., Curran S., Murray G.I. (2002). The structure, regulation, and function of human matrix metalloproteinase-13. Crit. Rev. Biochem. Mol. Biol..

[28] Bonacquisti E.E., Nguyen J. (2019). Connexin 43 (Cx43) in cancer: Implications for therapeutic approaches via gap junctions. Cancer Lett..

[29] Knäuper V., Will H., López-Otin C., Smith B., Atkinson S.J., Stanton H., Hembry R.M., Murphy G. (1996). Cellular mechanisms for human procollagenase-3 (MMP-13) activation. Evidence that MT1-MMP (MMP-14) and gelatinase a (MMP-2) are able to generate active enzyme. J. Biol. Chem..

[30] Aasen T., Mesnil M., Naus C.C., Lampe P.D., Laird D.W. (2016). Gap junctions and cancer: Communicating for 50 years. Nat. Rev. Cancer.

[31] Lv M., Shen Y., Yang J., Li S., Wang B., Chen Z., Li P., Liu P., Yang J. (2017). Angiomotin Family Members: Oncogenes or Tumor Suppressors?. Int. J. Biol. Sci..

[32] Tan I.A., Ricciardelli C., Russell D.L. (2013). The metalloproteinase ADAMTS1: A comprehensive review of its role in tumorigenic and metastatic pathways. Int. J. Cancer.

[33] Marimuthu A., Chavan S., Sathe G., Sahasrabuddhe N.A., Srikanth S.M., Renuse S., Ahmad S., Radhakrishnan A., Barbhuiya M.A., Kumar R.V. (2013). Identification of head and neck squamous cell carcinoma biomarker candidates through proteomic analysis of cancer cell secretome. Biochim. Biophys. Acta.

[34] Van der Flier A., Sonnenberg A. (2001). Structural and functional aspects of filamins. Biochim. Biophys. Acta.

[35] Knyazeva A., Khudiakov A., Vaz R., Muravyev A., Sukhareva K., Sejersen T., Kostareva A. (2020). FLNC Expression Level Influences the Activity of TEAD-YAP/TAZ Signaling. Genes.

[36] Chen Y.J., Chang J.T., Lee L., Wang H.M., Liao C.T., Chiu C.C., Chen P.J., Cheng A.J. (2007). DSG3 is overexpressed in head neck cancer and is a potential molecular target for inhibition of oncogenesis. Oncogene.

[37] Brown L., Waseem A., Cruz I.N., Szary J., Gunic E., Mannan T., Unadkat’ M., Yang M., Valderrama F., O’Toole E.A. (2014). Desmoglein 3 promotes cancer cell migration and invasion by regulating activator protein 1 and protein kinase C-dependent-Ezrin activation. Oncogene.

[38] Takahashi H., Iriki H., Asahina Y. (2023). T cell autoimmunity and immune regulation to desmoglein 3, a pemphigus autoantigen. J. Dermatol..

[39] Pollmann R., Schmidt T., Eming R., Hertl M. (2018). Pemphigus: A Comprehensive Review on Pathogenesis, Clinical Presentation and Novel Therapeutic Approaches. Clin. Rev. Allergy Immunol..

[40] Eming R., Hennerici T., Backlund J., Feliciani C., Visconti K.C., Willenborg S., Wohde J., Holmdahl R., Hertl M. (2014). Pathogenic IgG antibodies against desmoglein 3 in pemphigus vulgaris are regulated by HLA-DRB1*04:02-restricted T cells. J. Immunol..

[41] Li X., Ahmad U.S., Huang Y., Uttagomol J., Rehman A., Zhou K., Warnes G., McArthur S., Parkinson E.K., Wan H. (2019). Desmoglein-3 acts as a pro-survival protein by suppressing reactive oxygen species and doming whilst augmenting the tight junctions in MDCK cells. Mech. Ageing Dev..

[42] Rehman A., Cai Y., Hünefeld C., Jedličková H., Huang Y., Teck Teh M., Sharif Ahmad U., Uttagomol J., Wang Y., Kang A. (2019). The desmosomal cadherin desmoglein-3 acts as a keratinocyte anti-stress protein via suppression of p53. Cell Death Dis..

[43] Mannan T., Jing S., Foroushania S.H., Fortune F., Wan H. (2011). RNAi-mediated inhibition of the desmosomal cadherin (desmoglein 3) impairs epithelial cell proliferation. Cell Prolif..

[44] Troeltzsch M., Künzel V., Haidari S., Troeltzsch M., Otto S., Ehrenfeld M., Probst F., Knösel T. (2022). Desmoglein-3 overexpression in oral squamous cell carcinoma is associated with metastasis formation and early recurrence: An immunohistochemical study. J. Cranio-Maxillofac. Surg..

