# Supplementary figures and images for: Comparative Transcriptome Analysis Identifies Desmoglein-3 as a Potential Oncogene in Oral Cancer Cells
Source: Cells. 2023 Nov 26;12(23):2710. doi: 10.3390/cells12232710 (PMC10705960; doi:10.3390/cells12232710)

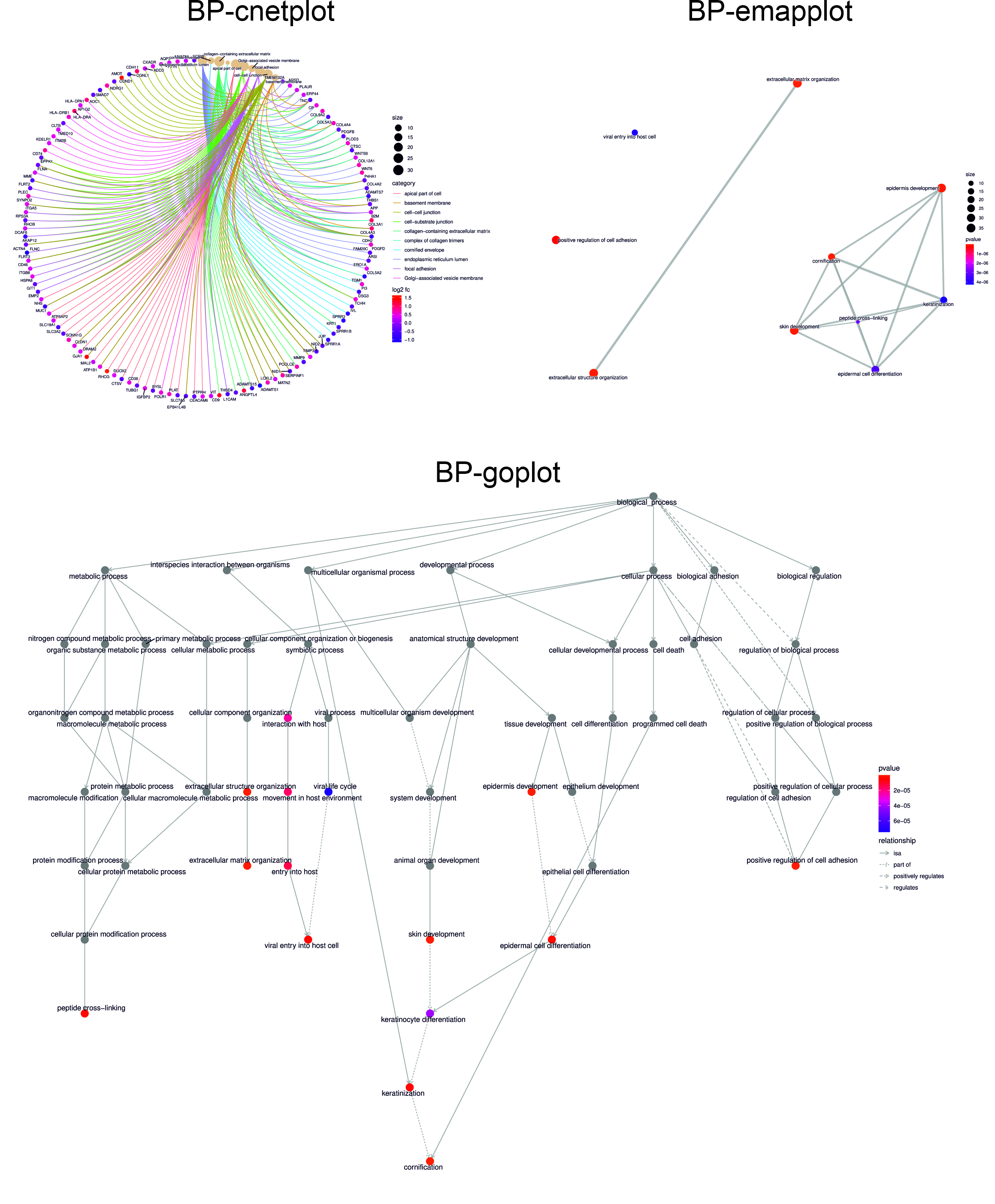

Supplement: Supplementary file 1 [file cells-12-02710-s001.zip › Fig S1.tif]

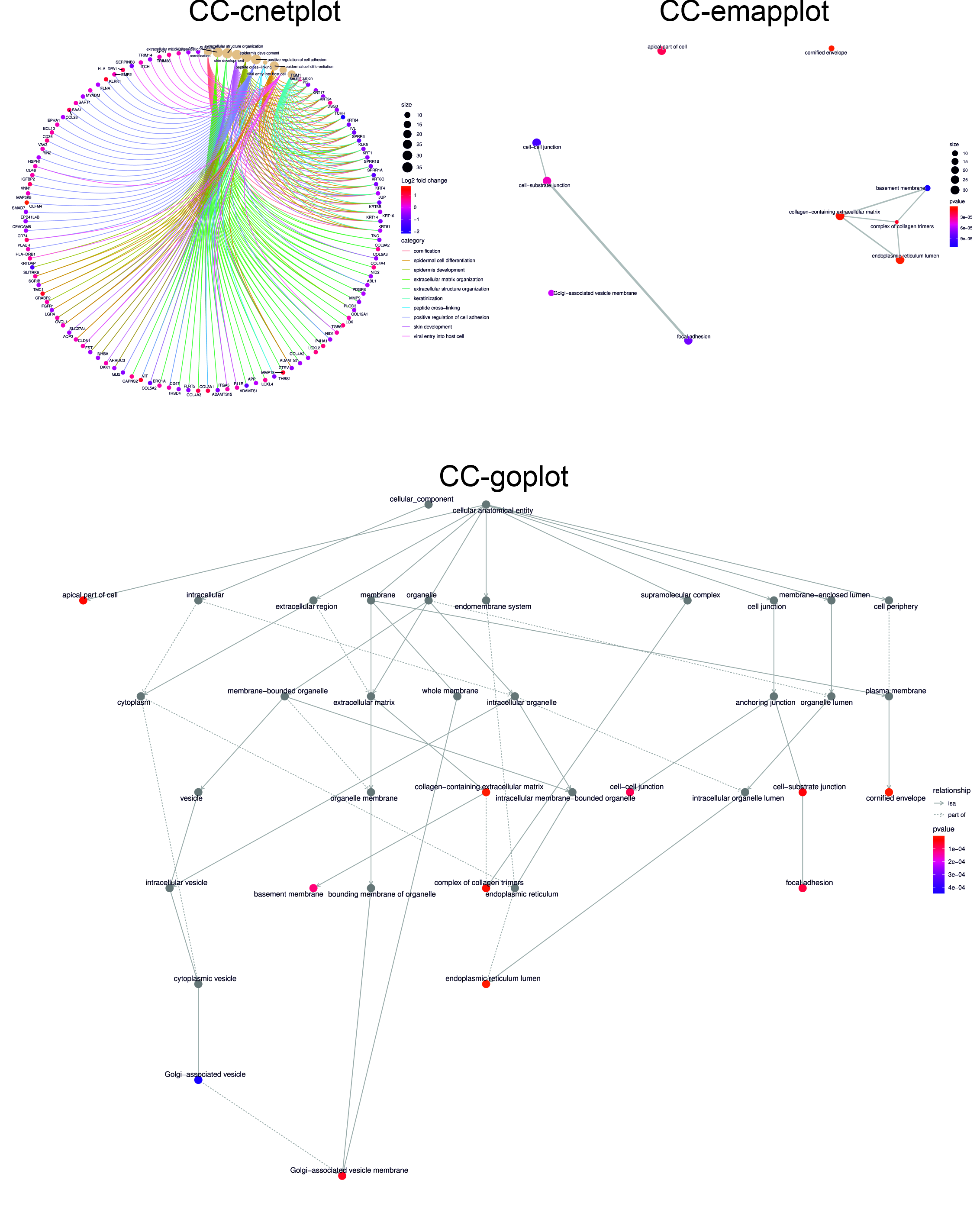

Supplement: Supplementary file 1 [file cells-12-02710-s001.zip › Fig S2.tif]

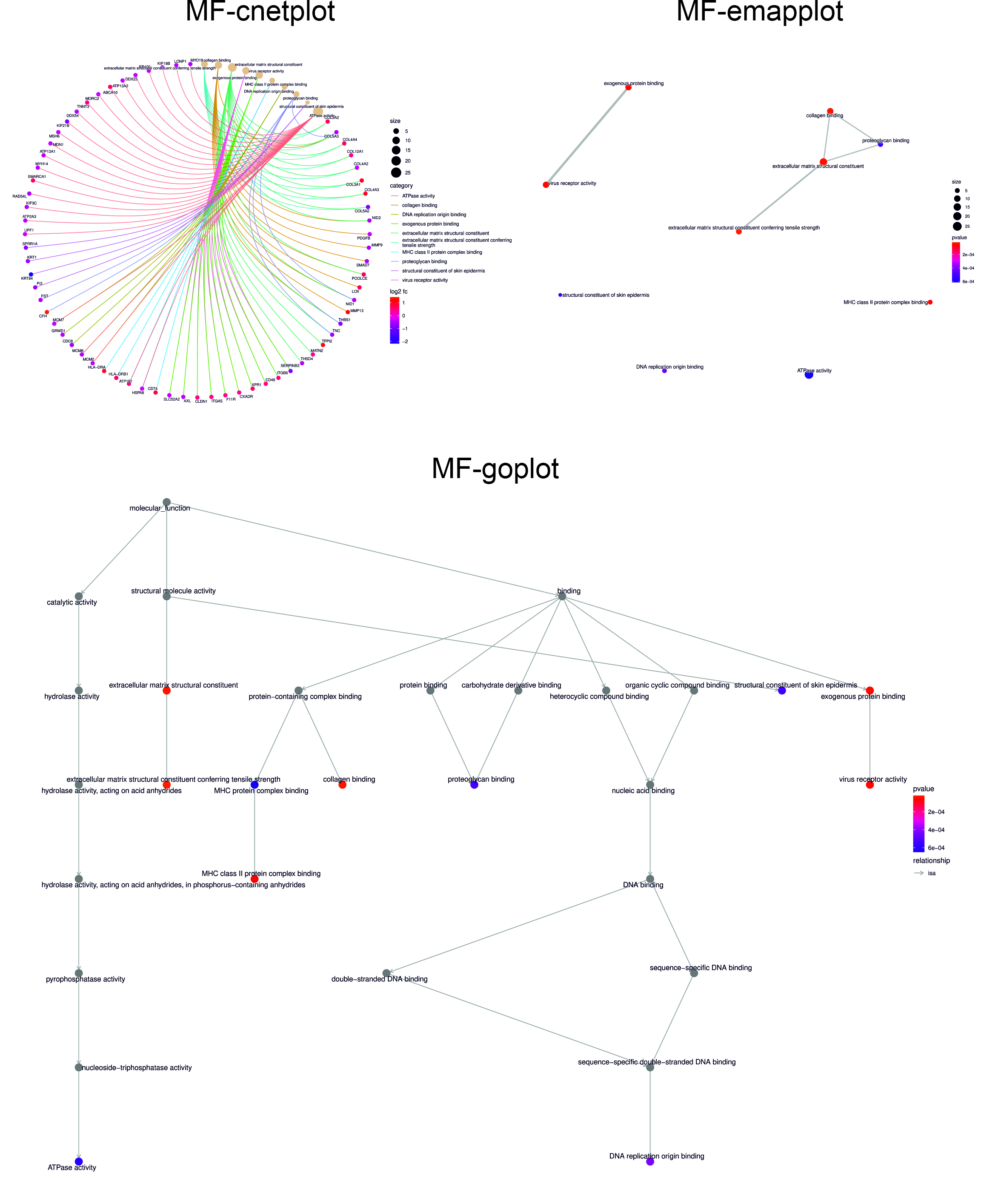

Supplement: Supplementary file 1 [file cells-12-02710-s001.zip › Fig S3.tif]
